# Supplementary material for: BMP signaling downstream of the Highwire E3 ligase sensitizes nociceptors
Source: PLoS Genet. 2018 Jul 12;14(7):e1007464. doi: 10.1371/journal.pgen.1007464 (PMC6042685; doi:10.1371/journal.pgen.1007464)
Supplement: S1 Fig — The hiwND8, hiwΔN and hiwΔC mutants all showed a longer latency to respond to a 46°C thermal probe than controls (w1118). n = 63, 46, 53 and 39 respectively. *** p < 0.001 (Steel’s test versus control). All error bars represent standard error. (PDF) [file pgen.1007464.s001.pdf]

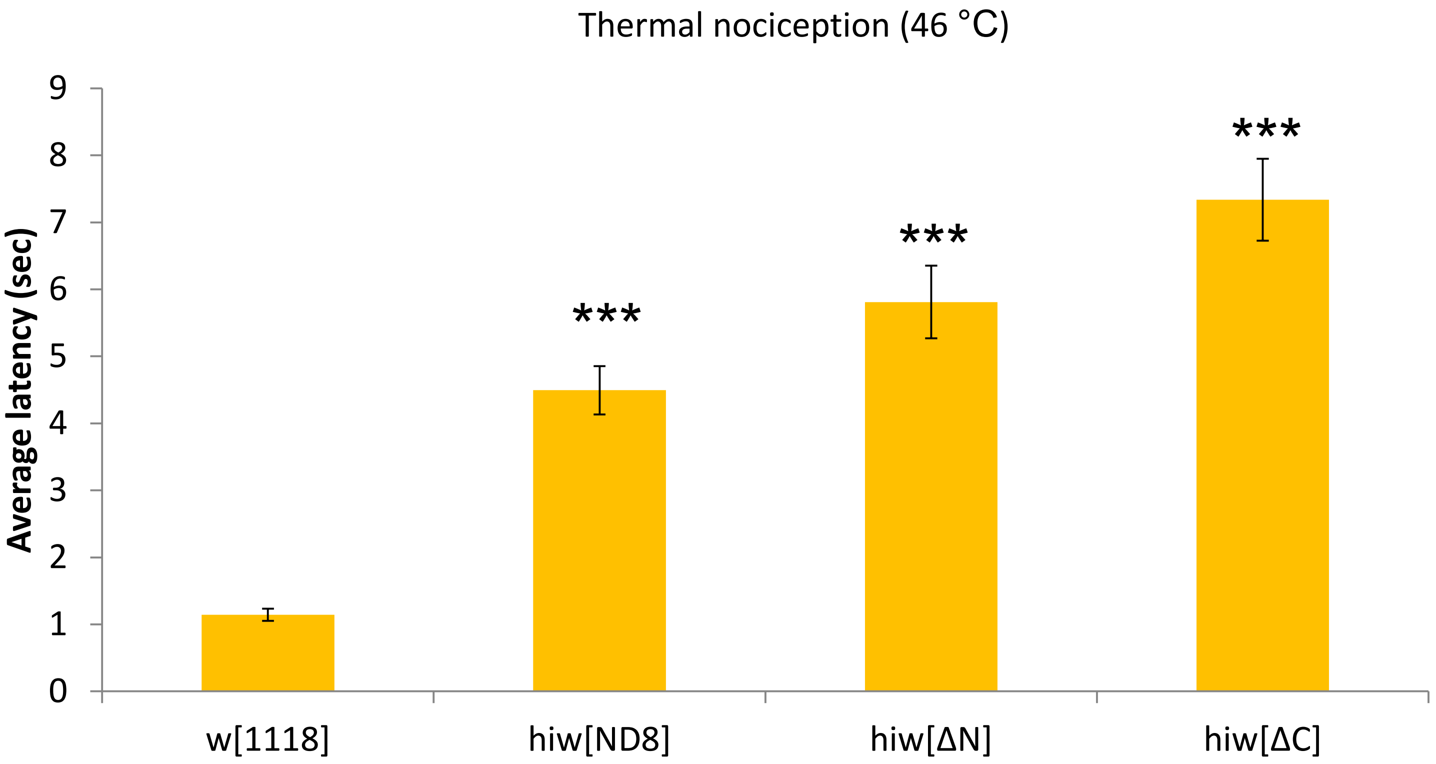

Figure S1. Multiple *hiw* alleles show insensitivity in thermal nociception. The hiw^{ND8}, $hiw^{\Delta N}$ *and* $hiw^{\Delta C}$ mutants all showed a longer latency to respond to a 46°C thermal probe than controls (w^{1118}). n = 63, 46, 53 and 39 respectively. *** $p < 0.001$ (Steel's test versus control). All error bars represent standard error.
