# Supplementary material for: BMP signaling downstream of the Highwire E3 ligase sensitizes nociceptors
Source: PLoS Genet. 2018 Jul 12;14(7):e1007464. doi: 10.1371/journal.pgen.1007464 (PMC6042685; doi:10.1371/journal.pgen.1007464)
Supplement: S2 Fig — UAS-hiw△Ring and UAS-tkvQD were crossed to w1118 and tested in thermal nociception. The UAS strains that caused thermal hypersensitivity when crossed to ppk-GAL4 did not show significant hypersensitive phenotypes when crossed to w1118 in comparison to controls (ppk-GAL4 x w1118). n = 95, 70 and 63. All error bars represent standard error. (PDF) [file pgen.1007464.s002.pdf]

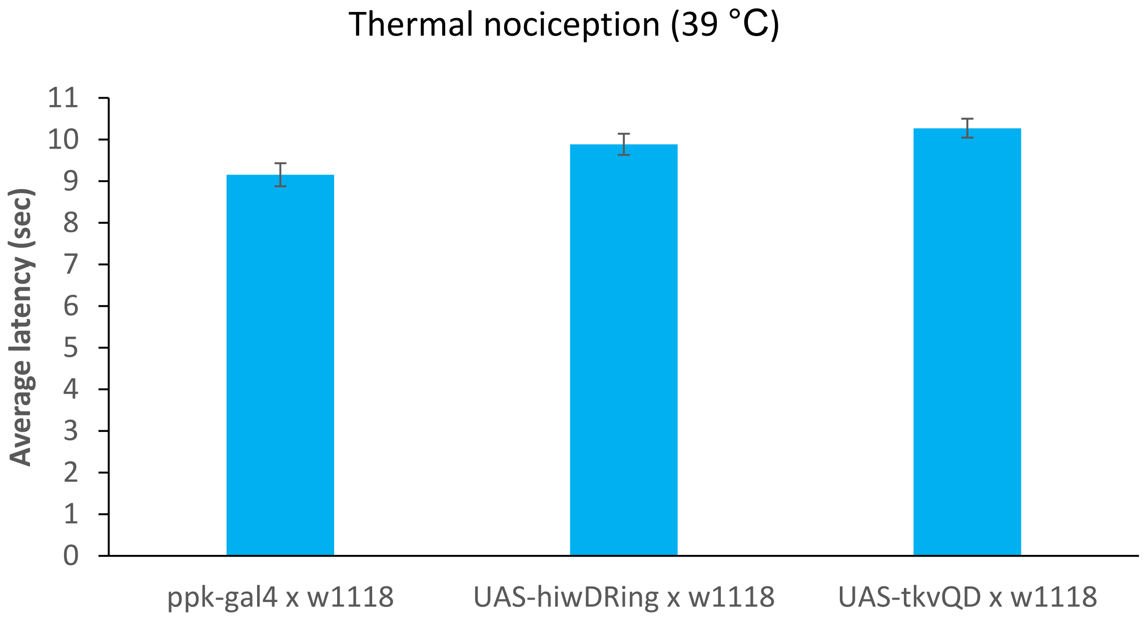

Figure S2. Phenotypes of UAS transgene were GAL4 driver-dependent. UAS-hiw△Ring and UAS-tkv^{QD} were crossed to *w*^{1118} and tested in thermal nociception. The UAS strains that caused thermal hypersensitivity when crossed to ppk-GAL4 did not show significant hypersensitive phenotypes when crossed to *w*^{1118} in comparison to controls (ppk-GAL4 x *w*^{1118}). n = 95, 70 and 63. All error bars represent standard error.
