# Supplementary material for: BMP signaling downstream of the Highwire E3 ligase sensitizes nociceptors
Source: PLoS Genet. 2018 Jul 12;14(7):e1007464. doi: 10.1371/journal.pgen.1007464 (PMC6042685; doi:10.1371/journal.pgen.1007464)
Supplement: S3 Fig — In comparison to controls (ppk-GAL4 x w1118, n = 115), animals expressing mad1 alone in nociceptors (ppk-GAL4 x UAS-mad1, n = 63, p > 0.99 versus control) and ones expressing hiwΔRing and mad in nociceptors (ppk-GAL4 UAS-hiwΔRing x UAS-mad1, n = 61, p > 0.14 versus control) did not show significant thermal insensitivity phenotype. All error bars represent standard error. (PDF) [file pgen.1007464.s003.pdf]

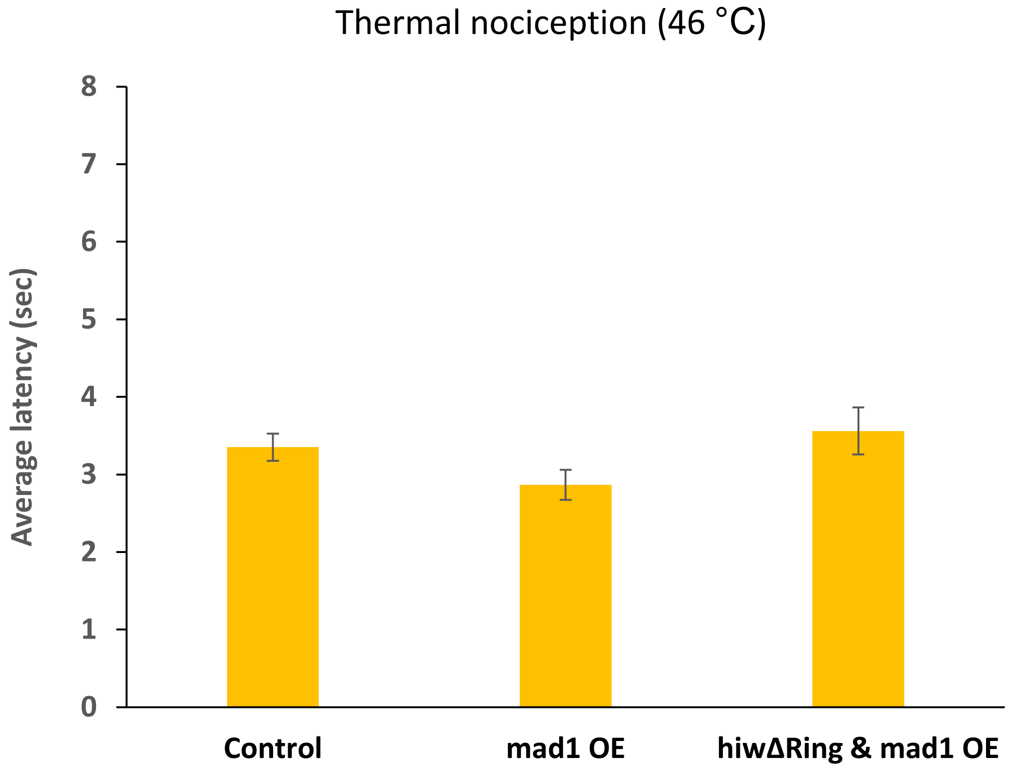

Figure S3. Expressing mad[1] alone or hiw△Ring and mad[1] did not cause thermal insensitivity .

In comparison to controls (ppk-GAL4 x *w*[1118], n = 115), animals expressing mad[1] alone in nociceptors (ppk-GAL4 x *UAS-mad[1]*, n = 63, p > 0.99 versus control) and ones expressing hiwΔRing and mad in nociceptors (ppk-GAL4 *UAS-hiwΔRing x UAS-mad[1]*, n = 61, p > 0.14 versus control) did not show significant thermal insensitivity phenotype. All error bars represent standard error.
