# Supplementary material for: BMP signaling downstream of the Highwire E3 ligase sensitizes nociceptors
Source: PLoS Genet. 2018 Jul 12;14(7):e1007464. doi: 10.1371/journal.pgen.1007464 (PMC6042685; doi:10.1371/journal.pgen.1007464)
Supplement: S4 Fig — (A-C) Representative pictures of pMad immunoreactivity in nociceptors of controls (ppk-GAL4 UAS-mCD8::GFP/+), wild-type wnd OE (ppk-GAL4 UAS-mCD8::GFP/UAS-wnd) and kinase-dead wnd (wndKD) OE (ppk-GAL4 UAS-mCD8::GFP/UAS-wndKD). (A’-C’) Split images for pMad signals. Scale bars represent 10 μm. (D) Quantified data for normalized nuclear pMad immunoreactivity. The overexpression of wild-type wnd showed 112 ± 9% increase of nuclear pMad signals, while that of kinase-dead version did not show a significant difference compared to controls. n = 12 for each genotypes. *** p < 0.001 (Steel’s test versus control). Error bars represent standard error. (PDF) [file pgen.1007464.s004.pdf]

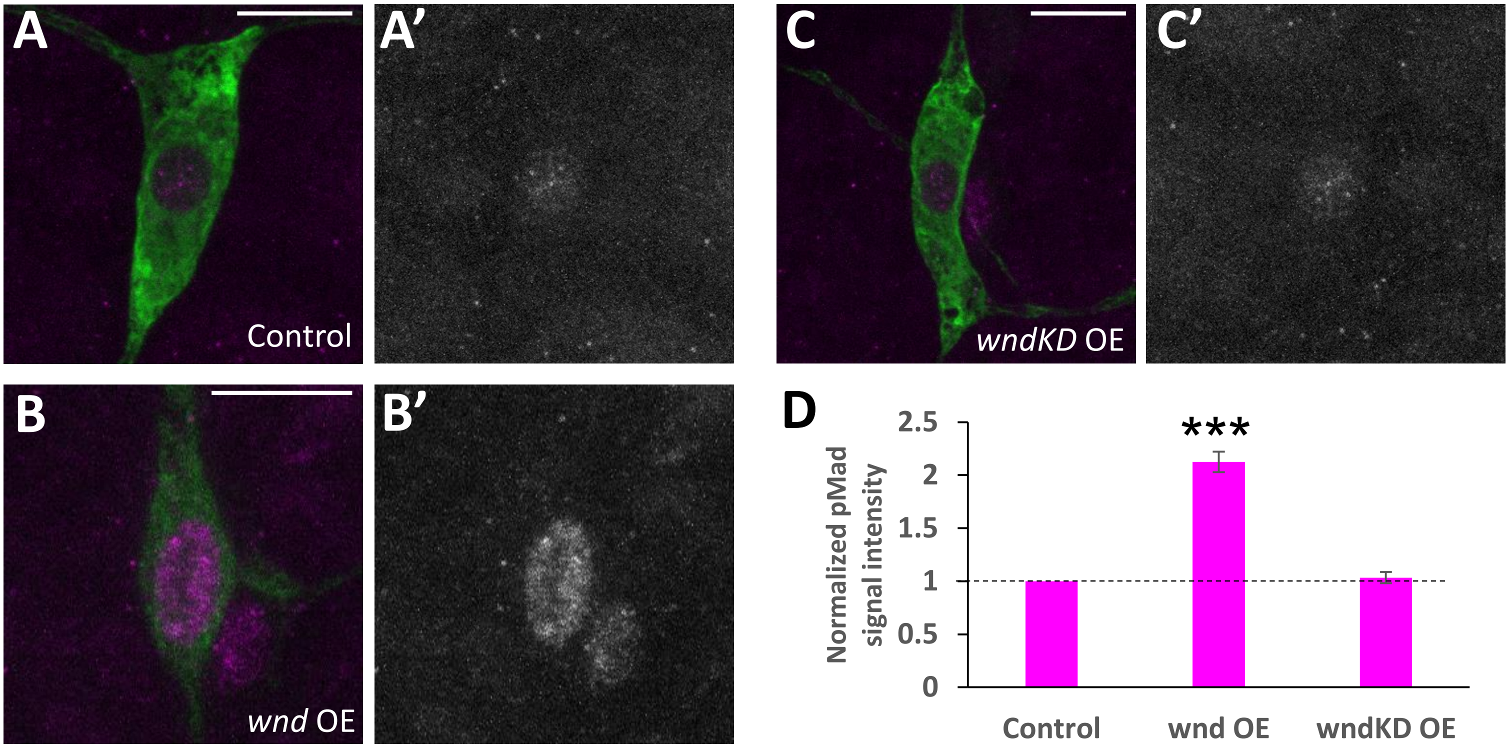

Figure S4. Overexpression of *wnd* leads to an up-regulation of nuclear pMad.

(A-C) Representative pictures of pMad immunoreactivity in nociceptors of controls (*ppk-GAL4 UAS-mCD8::GFP*/+), wild-type *wnd* OE (*ppk-GAL4 UAS-mCD8::GFP*/*UAS-wnd*) and kinase-dead *wnd* (wnd^{KD}) OE (*ppk-GAL4 UAS-mCD8::GFP*/$UAS\text{-}wnd^{KD}$). (A’-C’) Split images for pMad signals. Scale bars represent 10 µm. (D) Quantified data for normalized nuclear pMad immunoreactivity. The overexpression of wild-type *wnd* showed 112 ± 9% increase of nuclear pMad signals, while that of kinase-dead version did not show a significant difference compared to controls. n = 12 for each genotypes. *** $p < 0.001$ (Steel’s test versus control). Error bars represent standard error.
