# Supplementary material for: BMP signaling downstream of the Highwire E3 ligase sensitizes nociceptors
Source: PLoS Genet. 2018 Jul 12;14(7):e1007464. doi: 10.1371/journal.pgen.1007464 (PMC6042685; doi:10.1371/journal.pgen.1007464)
Supplement: S5 Fig — (A and B) Control animals (ppk-GAL4 UAS-mCD8::GFP; tub-GAL80ts x w1118) and tkvQD animals (ppk-GAL4 UAS-mCD8::GFP; tub-GAL80ts x UAS-tkvQD) with or without 22 hour heat induction were tested in thermal nociception. (A) After the 22 hour incubation at 30°C, both genotypes showed delayed responses to a 39°C thermal probe. (B) The percentage of larvae that showed NEL in response to the noxious heat stimulus in 10 seconds was also significantly lowered. n = 19, 24, 27, and 21. (C and D) Similar suppression of NEL in response to heat was also observed after 6 hour incubation at 30°C. n = 35, 33, 30, and 25. All error bars represent standard error. (PDF) [file pgen.1007464.s005.pdf]

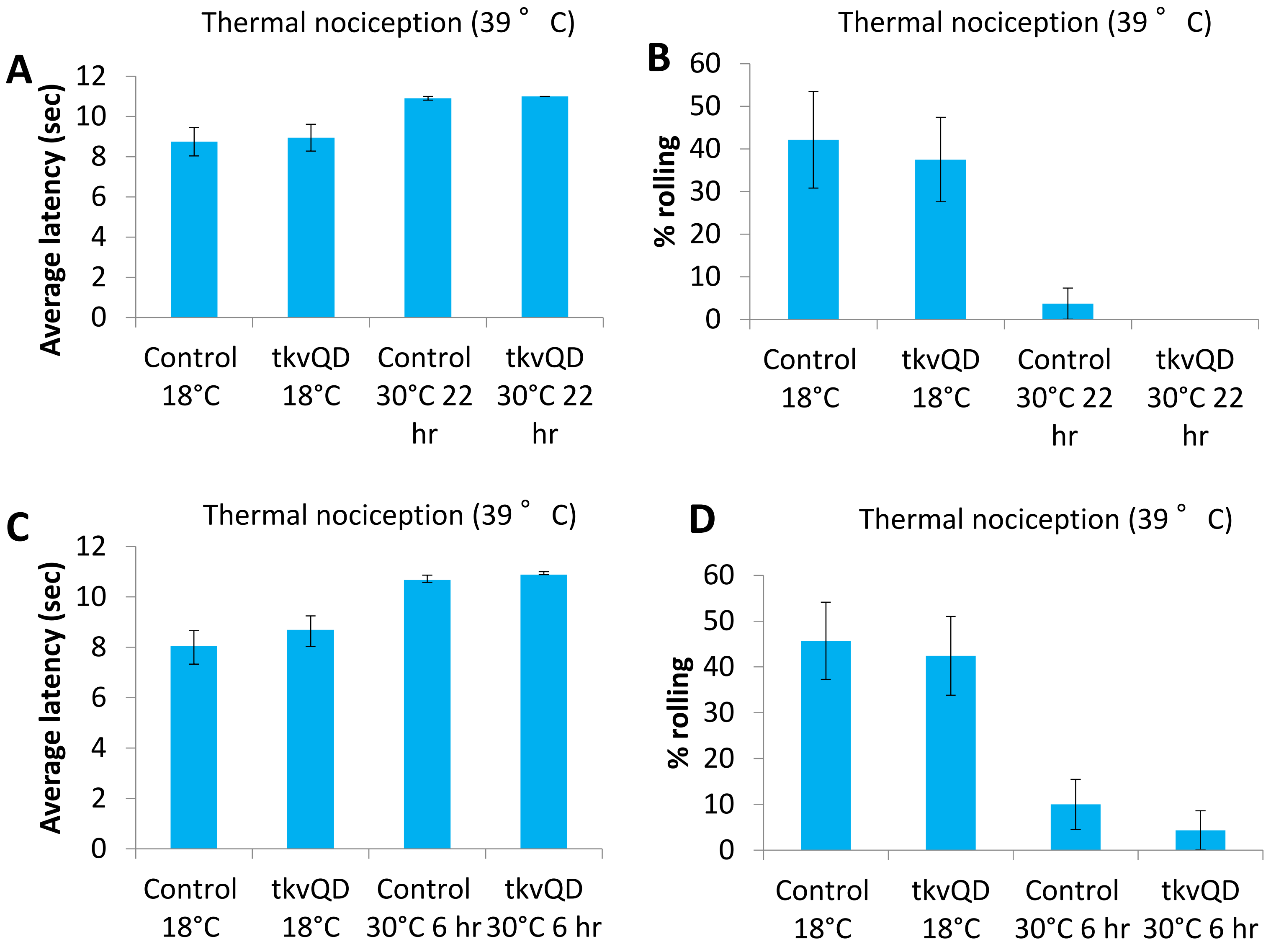

Figure S5. Incubation at 30° C causes insensitivity to noxious heat.
(A and B) Control animals (ppk-GAL4 UAS-mCD8::GFP; tub-GAL80^{ts} x w^{1118}) and *tkv^{QD}* animals (ppk-GAL4 UAS-mCD8::GFP; tub-GAL80^{ts} x UAS-*tkv^{QD}*) with or without 22 hour heat induction were tested in thermal nociception. (A) After the 22 hour incubation at 30° C, both genotypes showed delayed responses to a 39° C thermal probe. (B) The percentage of larvae that showed NEL in response to the noxious heat stimulus in 10 seconds was also significantly lowered. n = 19, 24, 27, and 21. (C and D) Similar suppression of NEL in response to heat was also observed after 6 hour incubation at 30° C. n = 35, 33, 30, and 25. All error bars represent standard error.
