# Supplementary material for: BMP signaling downstream of the Highwire E3 ligase sensitizes nociceptors
Source: PLoS Genet. 2018 Jul 12;14(7):e1007464. doi: 10.1371/journal.pgen.1007464 (PMC6042685; doi:10.1371/journal.pgen.1007464)
Supplement: S6 Fig — In control animals (ppk-GAL4 UAS-ChR2::YFP x w1118), males showed a lower responsiveness to optogenetic nociceptor stimulations compared to females. n = 191 and 211. All error bars represent standard error. (PDF) [file pgen.1007464.s006.pdf]

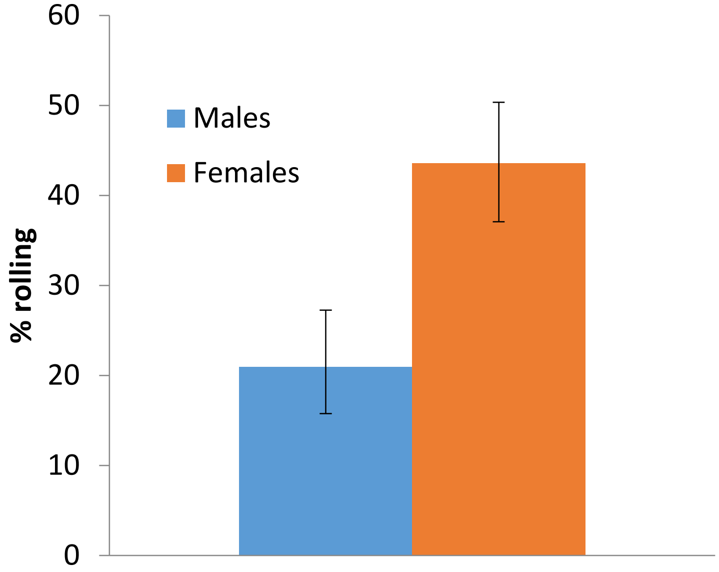

Figure S6. Sexual differences in the responsiveness to optogenetic nociceptor stimulations. In control animals (*ppk-GAL4 UAS-ChR2::YFP* x w^{1118}), males showed a lower responsiveness to optogenetic nociceptor stimulations compared to females. n = 191 and 211. All error bars represent standard error.
